# Barriers and Enablers to Routine Clinical Implementation of Cardiac Implantable Electronic Device Remote Monitoring in Australia Among Cardiologists, Cardiac Physiologists, Nurses, and Patients: Interview Study

**DOI:** 10.2196/67758

**Published:** 2025-07-18

**Authors:** Brodie Sheahen, Edel T O'Hagan, Kenneth Cho, Tim Shaw, Astin Lee, Sean Lal, Aaron L Sverdlov, Clara Chow

**Affiliations:** 1 Westmead Applied Research Center, The University of Sydney, Westmead, Australia, 61 488975865; 2 Charles Perkins Centre, The University of Sydney, Camperdown, Australia; 3 Cardiology Department, The Wollongong Hospital, Wollongong, Australia; 4 School of Medicine, Faculty of Science Medicine and Health, University of Wollongong, Wollongong, Australia; 5 Cardiology Department, Royal Prince Alfred Hospital, Sydney, Australia; 6 Cardiology Department, John Hunter Hospital, Newcastle, Australia; 7 Cardiology Department, Westmead Hospital, Westmead, Australia

**Keywords:** remote monitoring, cardiac implantable electronic devices, cardiac implant, patient engagement, barriers and enablers, cardiovascular disease, CVD, congestive heart failure, CHF, myocardial infarction, MI, unstable angina, angina, cardiac arrest, atherosclerosis, cardiology, cardiologist

## Abstract

**Background:**

Remote monitoring (RM) of cardiac implantable electronic devices (CIEDs) has demonstrated many patient and health care system benefits. Consequently, the use of RM technology for patients with CIEDs is the standard of care as highlighted by international guidelines. However, RM has not yet been integrated into universal, routine clinical practice.

**Objective:**

We aimed to establish key stakeholder perspectives on the barriers and enablers of CIED RM implementation and to apply the theoretical domain framework to highlight the most effective approaches to facilitate routine adoption of CIED RM.

**Methods:**

This was a qualitative study, using semistructured interviews to explore the barriers and enablers encountered when incorporating RM into CIED management. Participants included cardiologists, cardiac clinicians or physiologists, nurses, and patients. Interviews were transcribed verbatim and analyzed through inductive thematic analysis and deductive approaches using the NVivo (version 14; QRS International Pty Ltd) software. The theoretical domains framework was used to understand barriers and enablers. In the inductive phase, we did not assess trustworthiness, as our thematic analysis approach views data as interpretations rather than objective truths. In the deductive phase, we conferred to ensure consistency in theme alignment with existing frameworks.

**Results:**

Interviews were conducted among 35 participants (16 patients, 10 cardiologists, and 9 cardiac physiologists and nurses). We identified 5 main themes and their associated subthemes, with 1 representing an enabler and 4 representing barriers. They were: (1) patient benefits from RM, such as improved CIED and cardiovascular management, and improved patient-centered care; (2) insufficient allocation of CIED RM resources, which included insufficient RM clinic funding and staffing, insufficient RM service reimbursement, and RM infrastructure and access inequity; (3) suboptimal management of data, which includes inconsistent RM alert interpretation and management, lack of guidance for clinic staff on RM data management, and an increased alert burden for clinics; (4) insufficient patient education post-CIED implant, this was attributed to limited health care worker availability and resulted in inadequate patient CIED and RM knowledge postimplant and patient anxiety associated with RM; and (5) patient engagement with CIED management, which included the need for increased patient interaction with RM alerts and the ability to share data with patients. These subthemes were mapped to 6 specific domains of the theoretical domains framework: “Beliefs About Capabilities,” “Environmental Context and Resources,” “Beliefs About Consequences,” “Knowledge,” “Emotions,” and “Goals.”

**Conclusions:**

Patient engagement was identified in 3 of the 5 themes describing barriers and enablers to RM. These highlight the importance of addressing patient engagement with RM to better implement and integrate the use of RM into routine clinical practice. Barriers and enablers extend across multiple domains and suggest that a multipronged approach is required to translate the gold standard care of RM to routine clinical practice.

## Introduction

The use of remote monitoring (RM) is the standard of care for patients with cardiac implantable electronic devices (CIEDs) and is poised for wider adoption in the coming years, backed by growing endorsements from large cardiac societies such as the Heart Rhythm Society and Cardiac Society of Australia and New Zealand [1,2]. Whilst this uptake in RM is a positive move for improving patient care, in turn, it raises concerns about the capacity of device clinics to manage the associated workload [3,4]. Recent studies have estimated that managing 1000 patients with CIED with RM necessitates a workforce commitment of approximately 30‐46 hours per week by the clinical team [5].

The relative novelty of the technology creates challenges when incorporating CIED RM into clinical practice. Insufficient funding, lack of appropriate infrastructure, and lack of standardized workflow are commonly cited barriers [3,4,6,7]. Furthermore, despite some cardiac organizations placing a greater emphasis on patient engagement in the CIED, engagement initiatives are lacking, particularly surrounding patient education and information delivery [8,9]. The research to date suggests that implementation of RM requires cohesive management among many stakeholders, such as cardiologists, nurses, cardiac physiologists, and patients.

It is recognized across multiple sectors of health care that effective and sustainable implementation of research and innovations into clinical care relies on relevant stakeholders’ input into the integration of the intervention [10]. A comprehensive implementation analysis of RM across all relevant stakeholders has not been conducted internationally. Currently, there is a scarcity of information on stakeholder perspectives of the barriers and enablers of CIED RM. Thus, this study aimed to (1) establish broad stakeholder perspectives on issues surrounding the routine implementation of CIED RM and (2) apply the theoretical domain framework to highlight, through an implantation science lens, the most effective approaches to facilitate routine adoption of CIED RM.

## Methods

### Study Overview

This was a qualitative study, using semistructured interviews to explore individual perspectives on barriers and facilitators to RM of CIEDs and patient engagement. This study adhered to the COREQ (Consolidated Criteria for Reporting Qualitative Research) [11] checklist for study execution and subsequent reporting.

### Theoretical Domains Framework

We used the theoretical domains framework (TDF) to understand barriers and enablers through an implementation science lens. The TDF is comprised of 14 domains and 84 constructs to bring together many behavior-change theories. It was designed to bridge the gap between behavior-change theory and various medical disciplines, making it both accessible and applicable to a wide range of health care professionals [12].

### Research Team and Reflexivity

We adopted a hybrid approach, combining postpositivist principles and codebook thematic analysis [13]. This approach recognizes that knowledge is never fully objective but integrates procedures to ensure rigor. Consistent with this perspective, we acknowledge that all observations are shaped by the researcher’s perspectives, assumptions, and contexts, which are tentative and subject to revision. The research team was composed of cardiologists (CC, AL, SL, AS, and KC), a doctor-in-training and PhD student (BS), clinical researchers (ETO, CC, AL, SL, AS, and KC), and a digital health expert (TS). Researchers (BS, EO, CC, and TS) have experience conducting qualitative research, while clinician-researchers (CC, AL, SL, AS, and KC) have clinical cardiology experience. Interviews were conducted by the lead researcher (BS). Participants were aware that the interviewer was a PhD student and doctor-in-training; however, they had not met him prior to their interview.

### Study Setting and Recruitment

Between July 2022 and April 2023, we identified stakeholders (cardiologists, cardiac physiologists, nurses, and patients) who either used RM or were involved in analyzing and deciding on appropriate action for the data or alerts received via CIED RM. All stakeholders were based in Australia. Australia’s health care system combines Medicare**,** which provides universal public coverage, in parallel with private insurance for additional services. Stakeholders were recruited from 5 hospitals providing CIED and at least some RM services to urban and regional areas of New South Wales, Australia: Westmead, Wollongong, Royal Prince Alfred, Concord, and John Hunter. Stakeholder eligibility criteria included being 18 years or older and English speaking. Patient-specific criteria included currently having a CIED in-situ, which is undergoing RM. Cardiologist-specific criteria included being a consultant, public hospital or private practice-based, and managing at least one patient currently receiving RM. Cardiac physiologist and nurse-specific criteria included managing at least one patient currently receiving RM and public hospital or private practice-based.

### Procedure or Data Collection

Specific interview guides (Multimedia Appendix 1) were developed based on the stakeholders being interviewed (cardiologists, cardiac physiologists, nurses or allied health clinicians, and patients). The interview guides explored (1) stakeholder perspectives on the barriers and facilitators of CIED RM and (2) patient engagement with CIED and overall cardiovascular disease (CVD) management. Additionally, participant demographic data were collected verbally at the beginning of each interview. To develop the interview guides, we conducted a comprehensive literature review, identifying relevant studies on patient perspectives and existing interview guides used in similar studies. Interview guides were further refined after consulting with a cardiologist and conducting pilot interviews to ensure that questions were clear, comprehensive, and appropriate for the target audience. Potential clinical participants (cardiologists, cardiac physiologists, and nurses) were identified through snowball sampling conducted by the principal investigators and clinical staff from each site, then invited to participate either via email or in person. Patients were identified through convenience sampling by site clinicians and were invited to participate via phone call or in person. There were no dropouts, and all participants who were approached agreed to partake in the study. Participants consented either electronically or verbally prior to study commencement. All interviews were conducted either over telephone calls or in person at a CIED clinic with only the researcher present. The interview duration ranged from 15 to 45 minutes. We continued to conduct interviews until the researcher judged that the dataset was sufficiently rich to meaningfully address the research question, conducting 35 interviews in total. This number exceeds the sample adequacy range suggested by Hennick and Kaiser, supporting the sufficiency of our sample. Interviews were audio recorded and transcribed verbatim, without field notes being taken. Participants did not receive a copy of the transcript to review or provide feedback on study findings.

### Data Analysis

**Figure 1.**
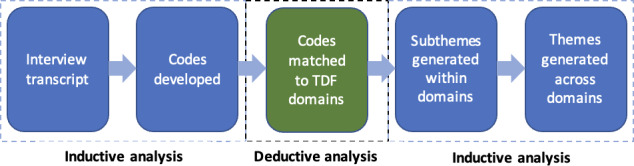
Thematic analysis of the interview data using an inductive and deductive approach. TDF: theoretical domains framework.

Interview transcripts were uploaded to NVivo (version 14; QRS International Pty Ltd) software. Two investigators (BS and EO) analyzed using a hybrid approach, combining the benefits of an inductive thematic analysis with a deductive approach [14] to represent the data in a generalizable way using the TDF (Figure 1).

Data were analyzed in an iterative process. Initially 2 researchers (BS and EO) read and reread the first 5 transcripts and coding fragments relevant to the research question. The codes were reviewed, discussed, and deliberated between investigators (BS and EO) to compare the data interpretation. The deliberation aimed to ensure we had comprehensively covered all aspects of the research question, to explore any potential nuances in the interpretation, and resulted in the initial codebook development. One investigator (BS) continued the analysis of the remaining transcripts. This process was continually reviewed with refined versions of the codebook reviewed by the investigator (EO). This process enabled a transparent and rigorous approach to coding while remaining sensitive to the inductive and interpretive nature of the analysis.

Using a deductive analysis approach, codes were then matched to the appropriate TDF domains. This process was reviewed, discussed, and deliberated between investigators (BS and EO) until consensus was reached and consistent.

One investigator (BS) used an inductive analysis approach to develop subthemes from the codes before developing overarching themes [15]. Themes and subthemes were generated from codes across all participants, rather than stratifying by stakeholder title (cardiologists, cardiac physiologists, nurses, and patients). This process was reviewed and discussed between investigators (BS and EO) until a consensus was reached, resulting in the final data output.

### Trustworthiness

In the inductive phase, we ensured rigor by using structured codebooks and multiple coders to independently code the same data. The coders discussed their interpretations to refine and align them, ensuring consistency in the analysis while preserving the interpretive flexibility of the approach. In contrast, in the deductive phase, we applied the TDF to categorize themes. To ensure consistency and coherence in this process, we compared interpretations and reached a consensus on domain alignment. This collaborative approach helped enhance the reliability of our deductive analysis while respecting the interpretive nature of qualitative research.

### Ethical Considerations

Ethics approval was granted by the Western Sydney Local Health District (2022/ETH00271). All participants provided informed consent to partake in the study prior to data collection and were informed that they could withdraw from the study at any time. Participants were assigned a study ID and had all data deidentified. No form of compensation was provided to any participant for their involvement in the study.

## Results

### Overview

A total of 35 interviews were conducted between July 2022 and April 2023. In total, 16 of the interviews were conducted with patients, 10 with cardiologists, and 9 with cardiac physiologists and cardiac nurses. The mean patient age was 73.1 (SD 10.7) years, and the majority were male (n=12, 75%) and born in Australia (n=12, 75%). Pacemakers (n=8, 50%) were the most common CIED type, and the mean duration of RM was 4.3 (SD 2.6) years. The mean cardiologist age was 46.2 (SD 6.3) years, and the majority were male (n=9, 90%), subspecialized in electrophysiology (n=7, 70%), had a mean duration of 12.3 (SD 6.6) years as a cardiologist, and a mean duration of 7.7 (3.6) years managing patients with RM. The mean physiologist or nurse age was 36.6 (SD 9.4), and the majority were female (n=5, 56%), and had a mean duration of 4.3 (SD 2.6) years managing patients with RM. Participant demographic and clinical experience results are presented in Table 1.

**Table 1.** Demographic, CIED^a^, and RM^b^ characteristics of interviewed stakeholders.

Characteristic	Value (n=35)
Patients (n=16)	
Age (years), mean (SD)	73.1 (10.7)
Male, n (%)	12 (75)
Country of birth, n (%)	
Australia	12 (75)
England	3 (19)
Lebanon	1 (6)
CIED indication, n (%)	
Ventricular tachycardia primary prevention	7 (44)
Atrial fibrillation	3 (19)
Bradycardia	3 (19)
Syncope	1 (6)
Arrhythmia (unknown to the patient)	2 (12)
CIED type, n (%)	
Pacemaker	8 (50)
Defibrillator	5 (31)
Cardiac resynchronization therapy—pacemaker	3 (19)
Duration receiving RM (years), mean (SD)	4.3 (2.6)
Physiologists or nurses (n=9)	
Age (years), mean (SD)	36.6 (9.4)
Male, n (%)	4 (44)
Location, n (%)	
Western Sydney	5 (56)
Illawarra	1 (11)
Newcastle	2 (22)
Sydney	1 (11)
Duration managing RM (years), mean (SD)	6.1 (2.6)
Cardiologists (n=10)	
Age (years), mean (SD)	46.2 (6.3)
Male, n (%)	9 (90)
Location, n (%)	
Western Sydney	3 (30)
Illawarra	1 (1)
Newcastle	3 (3)
Sydney	3 (3)
Cardiologist subspecialty, n (%)	
Electrophysiologist	7 (70)
Heart failure specialist	2 (20)
Proceduralist	1 (10)
Duration as cardiologist (years), mean (SD)	12.3 (6.6)
Duration managing RM (years), mean (SD)	7.7 (3.6)

a CIED: cardiac implantable electronic device.

b RM: remote monitoring.

**Figure 2.**
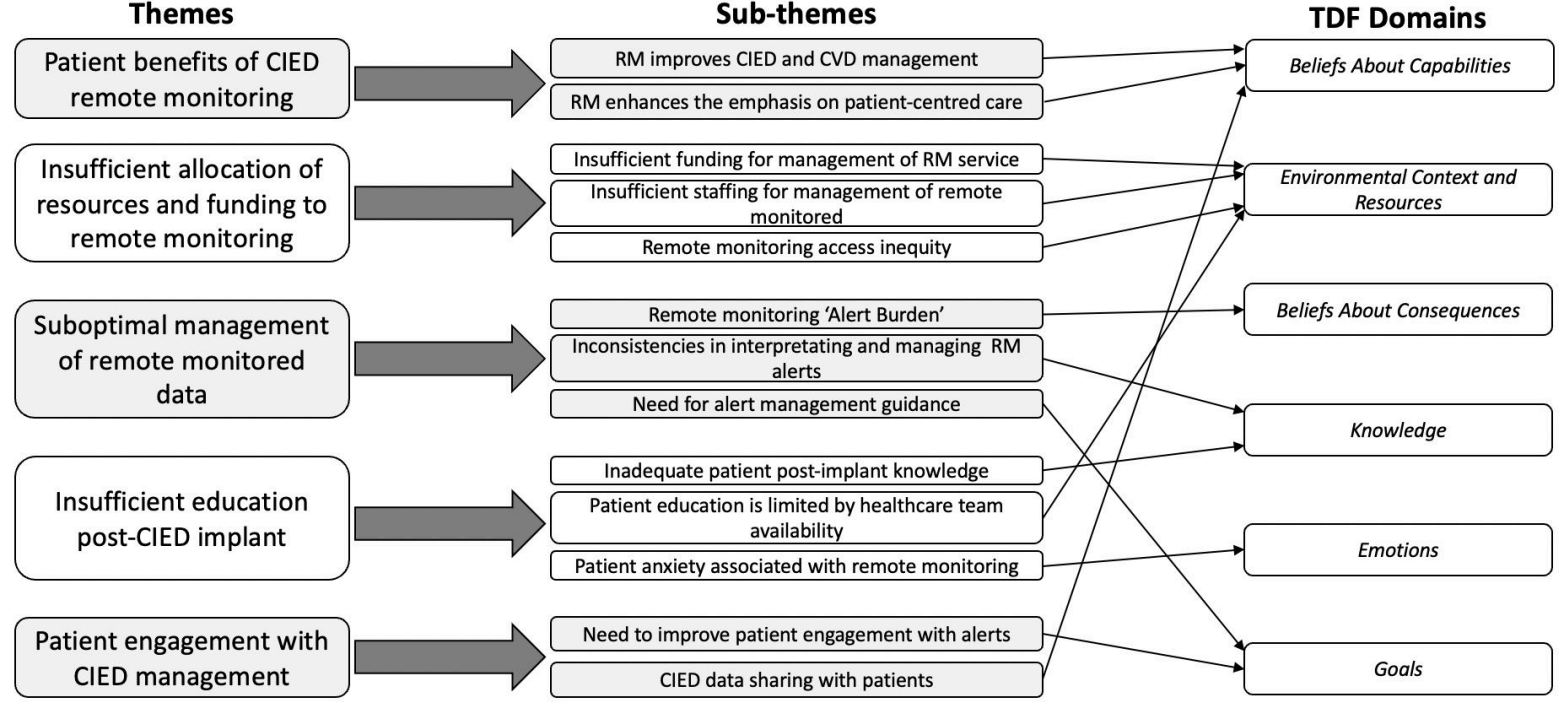
Themes and subthemes emerged from qualitative thematic analysis with allocation to the relevant TDF domains. CIED: cardiac implantable electronic device; CVD: cardiovascular disease; TDF: theoretical domains framework.

We organized our results into themes and subthemes. Themes and subthemes are summarized in Figure 2, with subthemes and codes described below. One theme was deemed an enabler, and 4 barriers to RM. Illustrative quotes for each subtheme and code are presented in Tables S1-S13 in Multimedia Appendix 2.

### Theme 1: Patient Benefits on RM

#### RM Improves CIED and CVD Management

The main benefits noted by stakeholders included the improved patient treatment outcomes facilitated by RM (Quotes 1-3 in Table S1 in Multimedia Appendix 2). These benefits were perceived to be largely driven by earlier detection of clinical issues (Quotes 4-9 in Table S1 in Multimedia Appendix 2), reduced postimplant issues (Quotes 10 and 11 in Table S1 in Multimedia Appendix 2), prevented hospital admissions (Quote 12 in Table S1 in Multimedia Appendix 2), and deployment of a service to rural and remote patients who otherwise have restricted access to CIED care (Quote 13 in Table S1 in Multimedia Appendix 2). Furthermore, clinicians reported that RM-based care enabled CIED management to be provided to patients without face-to-face review during the COVID-19 pandemic (Quote 14 in Table S1 in Multimedia Appendix 2).

#### RM Enhances the Emphasis on Patient-Centered Care

Cardiologists noted RM processes are designed to be user-friendly for patients (Quote in Table S2 in Multimedia Appendix 2). Physiologists highlighted that RM facilitates improved care for patients in nursing homes, who previously had difficulties attending face-to-face clinics (Quote 2 in Table S2 in Multimedia Appendix 2). Patients expressed gratitude for the reduced hospital visits required for CIED reviews (Quote 3 in Table S2 in Multimedia Appendix 2). Additionally, patients reported a sense of safety derived from having the health care team monitor their data through RM (Quotes 4 and 5 in Table S2 in Multimedia Appendix 2).

### Theme 2: Insufficient RM Resources, Funding, and Recognition of Workload and Skills

#### Funding for Management of RM Service

Barriers to the implementation and management of RM were centered around inadequate funding for clinics within the public sector. Cardiologists and physiologists reported that current reimbursement schemes fail to recognize the extensive tasks involved in providing the RM service and, in turn, do not provide adequate funding to deliver the service for improved patient care (Quotes 1-7 in Table S3 in Multimedia Appendix 2). Currently, clinicians reported that the delivery of RM comes with additional costs to the CIED clinics (Quote 8 in Table S3 in Multimedia Appendix 2), with some public hospitals reluctant to cover these costs despite the patient benefits (Quote 9 in Table S3 in Multimedia Appendix 2). Due to the inadequate funding, some clinicians reported they are unable to employ adequate staff to manage RM alerts (Quote 10 in Table S3 in Multimedia Appendix 2). Improved funding, infrastructure, and recognition by health services were recommended for RM development (Quotes 11 and 12 in Table S3 in Multimedia Appendix 2).

#### Staffing for Management of RM Alerts

Interpreting and responding to alerts can be time-consuming due to the range of “invisible” tasks required, which include, but are not limited to, confirming the alert accuracy, reviewing previous alerts, patient history and medications, patient contact, reprogramming, education, report development, and cardiologist escalation. The time to complete these tasks varies among physiologists based on their experience and confidence levels (Quote 1 in Table S4 in Multimedia Appendix 2). Physiologists mentioned that there is an inadequate number of staff employed to manage the RM workload (Quotes 2-4 in Table S10 in Multimedia Appendix 2), which can result in alerts not being managed in a timely fashion (Quotes 5 in Table S4 in Multimedia Appendix 2). Cardiologists mentioned that physiologists need more allocated time to manage alerts and scheduled reviews (Quotes 6 in Table S4 in Multimedia Appendix 2). Additionally, some cardiologists reported not having the capacity to review RM alerts (Quote 7 in Table S4 in Multimedia Appendix 2).

#### RM Access Inequity

Not all patients receive RM, and factors associated with receiving RM drive inequity in access (Quotes 1 and 2 in Table S5 in Multimedia Appendix 2). Cardiologists identified some of these factors: public payment models are poorly suited to the provision of RM, existing health services may not provision RM support, and smaller services may not have the skill mix to support RM (Quotes 3 and 4 in Table S5 in Multimedia Appendix 2). Additionally, smaller cardiology clinics often lack the necessary resources and capacity to offer the service (Quote 5 in Table S5 in Multimedia Appendix 2). Other factors hindering the equitable distribution of RM include the incompatibility of CIED, with many older models unable to support this technology (Quote 6 in Table S5 in Multimedia Appendix 2), and inadequate patient internet access, particularly affecting rural patients (Quote 7 in Table S5 in Multimedia Appendix 2).

### Theme 3: RM Data Management Burden and Risks

#### RM “Alert Burden”

Reviewing and managing alerts transmitted through RM was reported to be a time-consuming process for CIED clinic staff due to the range of “invisible” clinical and nonclinical tasks associated with alert receipt (Quotes 1-3 in Table S6 in Multimedia Appendix 2). Physiologists partly attributed this alert burden to their inability to modify alert parameters due to manufacturer system restrictions (Quotes 4 and 5 in Table S6 in Multimedia Appendix 2). Additionally, the transmission of alerts that are false positives further amplifies the workload for physiologists, which will only worsen with increasing CIED implants and RM utilization (Quote 6 in Table S6 in Multimedia Appendix 2). Consequently, the heightened alert burden resulting from a generalized alert setup and increased workload may compromise patient care and raise the likelihood of overlooking critical alerts (Quotes 7 and 8 in Table S6 in Multimedia Appendix 2).

#### Inconsistencies in Interpreting and Managing RM Alerts

Physiologists raised that there is a lack of uniformity in knowledge, skills, experience, and training to manage RM alerts (Quotes 1-3 in Table S7 in Multimedia Appendix 2). It was noted that in some countries, the cardiac physiologist workforce regulation requires registration with a Clinical Physiologists Registration Board, but in other countries like Australia, this is not mandatory. It was also raised that the lack of more specific clinical guidelines, or pragmatic training on responding and managing RM alerts, presents risks and challenges to service delivery (Quotes 4 and 5 in Table S7 in Multimedia Appendix 2). Further participants highlighted that there were significant differences among cardiologists and cardiology services in the appropriate management of RM alerts (Quote 6 in Table S7 in Multimedia Appendix 2), including what information is relevant to convey by physiologists to clinicians upon alert detection (Quote 7 in Table S7 in Multimedia Appendix 2). Furthermore, cardiologists highlighted a lack of standardization in the “baseline” settings of alert thresholds (Quote 8 in Table S7 in Multimedia Appendix 2).

#### Need for Alert Management Guidance

To enhance RM data management efficiency, physiologists have emphasized the need for RM alert management guidelines to provide support to CIED clinic staff (Quotes 1 and 2 in Table S8 in Multimedia Appendix 2). Additionally, cardiologists emphasize the importance of eliminating nonessential activities and implementing a process to receive alerts only for relevant, actionable issues (Quote 3 in Table S8 in Multimedia Appendix 2). Furthermore, cardiologists have expressed the need for a national consensus statement from experts in the RM field to provide standardized care for alert management (Quote 4 in Table S8 in Multimedia Appendix 2). Some clinics have taken the initiative to develop their internal alert management protocols, resulting in a reduction of “unnecessary*”* alerts and an overall decrease in workload (Quotes 5 and 6 in Table S8 in Multimedia Appendix 2).

### Theme 4: Insufficient Patient Education and Understanding of CIED and RM

#### Inadequate Patient Postimplant Knowledge

Patients mentioned that the information provided post-CIED implant was inadequate for their needs. Key areas of knowledge deficit upon discharge included a poor understanding of the RM service (Quote 1 in Table S9 in Multimedia Appendix 2) and a poor understanding of restrictions to daily activities (Quotes 2-7 in Table S9 in Multimedia Appendix 2). A barrier to effective patient education can be the timing of information delivery, with patients reporting being overwhelmed peri-implant and struggling to retain information (Quote 8 in Table S9 in Multimedia Appendix 2). In addition, discrepancies in information delivery exist between CIED types, with physiologists reporting that patients with implantable cardioverter defibrillator routinely receive greater education than patients with permanent pacemaker (Quote 9 in Table S9 in Multimedia Appendix 2). Furthermore, discrepancies exist based on insurance status, with private patients often receiving greater information than public patients (Quotes 10 and 11 in Table S9 in Multimedia Appendix 2). Following hospital discharge, patients reported that there is a lack of resources to acquire information (Quote 12 in Table S9 in Multimedia Appendix 2) and a lack of communication channels to ask specific questions (Quote 13 in Table S9 in Multimedia Appendix 2). Ultimately, both patients and physiologists acknowledge that there is no formal postdischarge program available to provide ongoing patient education and support, which in the future is something that is required for RM progression (Quotes 14-17 in Table S9 in Multimedia Appendix 2).

#### Patient Education is Limited by Health Care Team Availability

Cardiologists acknowledged that discussions with patients and the delivery of “proper” education do not often occur, largely due to workload and time constraints (Quotes 1-2 in Table S10 in Multimedia Appendix 2). Both patients and physiologists believe insufficient explanations and education are provided to patients upon scheduled reviews (Quotes 3 and 4 in Table S10 in Multimedia Appendix 2). Patients frequently mentioned that they often have questions regarding their care and restrictions; however, they do not have access to the health care team to ask these questions (Quotes 5 and 6 in Table S10 in Multimedia Appendix 2).

#### Patient Anxiety Associated With RM

The use of RM could be associated with heightened patient anxiety, influenced by various factors. Cardiologists noted that patients may be hesitant to embrace the RM service, primarily due to concerns about the privacy of their data (Quote 1 in Table S11 in Multimedia Appendix 2). Patients reported that they experienced increased anxiety when receiving inconsistent information regarding their data, such as the battery life of their CIED (Quote 2 in Table S11 in Multimedia Appendix 2). In addition, patients reported that travel-related scenarios would exacerbate their anxiety, with patients and their families expressing mistrust in both the CIED and the RM system when traveling and not having close access to a hospital (Quotes 3 and 4 in Table S11 in Multimedia Appendix 2). This mistrust has stemmed from inconsistencies in patient explanations of CIED clinic and RM capabilities.

### Theme 5: Patient Engagement

#### Need to Improve Patient Engagement With Alerts

Patients and cardiologists mentioned the need for improved communication with patients following alert detection (Quotes 1-3 in Table S12 in Multimedia Appendix 2). However, patient contact should only occur if the alerts are actionable and relevant to the patient (Quotes 4 and 5 in Table S12 in Multimedia Appendix 2). Patients and physiologists mentioned the benefit of using a digital tool such as an SMS text messaging platform or app to contact patients regarding alerts and for patients to ask questions (Quotes 6-8 in Table S12 in Multimedia Appendix 2).

#### CIED Data Sharing With Patients

There were varying perspectives on the provision of CIED data to patients. Cardiologists felt that patients should be able to access their CIED data (Quote 1 in Table S13 in Multimedia Appendix 2) and that personalized in-time data provided to the patient would improve engagement (Quotes 2 and 3 in Table S13 in Multimedia Appendix 2). However, nurses and physiologists anticipate that data sharing could increase patient anxiety and concern (Quotes 4-6 in Table S13 in Multimedia Appendix 2). Patients noted that if they were to have access to their data, there would have to be careful consideration of what was presented (Quote 7 in Table S13 in Multimedia Appendix 2), and suggested that the data would need to be delivered in a user-friendly format (Quotes 8 and 9 in Table S13 in Multimedia Appendix 2).

### TDF

Subthemes were categorized into 6 TDF domains. The subthemes “RM improves CIED and CVD management,” “RM enhances the emphasis on patient-centered care,” and “CIED data sharing with patients” were developed within the Beliefs About Capabilities domain. Subthemes “Insufficient funding for management of RM service” “Insufficient staffing for management of remote monitored,” and “Remote monitoring access inequity” were developed within the Environmental Context and Resources domain. The subtheme “Remote monitoring alert burden” was developed within the Beliefs About Consequences domain. Subthemes “Inconsistencies in interpreting and managing RM alerts” and “Inadequate patient postimplant knowledge” were developed within the Knowledge domain. The subtheme “Patient anxiety associated with remote monitoring” was developed within the Emotions domain. Finally, the subthemes “Need for alert management guidance” and “Need to improve patient engagement with alerts” were developed within the Goals domain.

## Discussion

### Principal Results

RM of CIEDs offers significant advantages for individuals with CVD; however, there is still a large scope for improved implementation. This study provides a current multidisciplinary perspective on RM implementation and a framework of barriers and enablers to address for improving future implementation and scale-up. We identified 5 main themes representing the barriers and facilitators to CIED with RM use. These themes are mapped to 6 domains of the TDF, which can inform targeted interventions to enhance implementation and maximize the potential benefits of CIED RM.

### Comparison With Other Work

Across the themes, there was a reinforcement of the benefits of CIED RM directly to the patient in both improved efficiencies in health care delivery and improved health outcomes through early detection of issues, prevention of hospital admissions, and better provision of care to rural or remote patients. These perspectives are corroborated by several recent studies which have demonstrated that RM enables earlier detection of actionable alerts [16], improves outcomes including reduced inappropriate shocks [17], decreases rates of strokes [16], and reduces mortality rates demonstrated in the pooled analysis of 3 RCTs using continuous RM [18]. Furthermore, improvements in health care service utilization have been demonstrated with reduced emergency department presentations [19], hospital admissions [20,21], and hospitalization length-of-stay times [21]. However, in patients with heart failure, RM has not consistently demonstrated benefits in mortality and heart failure hospital readmissions [22].

In total, 3 of the 5 themes identified centered on patient engagement, understanding, and perceived utility. Across subthemes, it was identified that RM enhances the focus on patient-centered care (offering a user-friendly service, minimizing in-person reviews, correlating concerns with CIED data, and extending the service to patients who would otherwise lack such care) and enhances the patient’s sense of care. This is underscored by expressions of patient satisfaction, appreciation, reassurance, and an improved sense of safety in managing their CIED and CVD. These observations align with prior studies that have consistently shown positive outcomes in terms of patient satisfaction [23,24], acceptance [25], and an enhanced feeling of safety [23,24,26].

However, resourcing and an inadequate recognition of the tasks arising from RM, as well as the skills and training needed to manage alerts, were consistently identified as barriers to CIED RM. Lack of funding and appropriate reimbursement schemes have also been seen as a prominent barrier in European and North American countries [6,27]. While a recent meta-analysis has demonstrated that CIED RM is a cost-effective intervention for health care systems [28], current models of care do not yet account for the additional tasks that arise from RM implementation, particularly those associated with alert management. Staff described alert management as comprising multiple additional phone calls, troubleshooting connectivity issues, alert triage, and scheduling in-person reviews [29]. Many staff and health services are not recognized for the increased workload associated with RM [27], which may be expected to rise with the increasing complexity of CVD, the complexity of technology, and the number of CIED implants.

RM data management was also consistently identified as a challenge to RM implementation. The “alert burden” associated with nonclinically significant alerts was particularly called out as a process management challenge. Contributing to this was the generalized nature of alert parameters, the discrepancies between alert interpretation, and the lack of clinical appropriateness guidance. Potential risks could also arise if the “alert burden” arising from “nonactionable” alerts jeopardizes patient care through the missing of time-critical alerts, a phenomenon described as “alert fatigue” [30]. Consequently, clinicians have expressed the need for the standardization of RM data management from the guidance of a national expert consensus panel. This call for RM standardization processes is not novel to this study, with multiple recent studies identifying the growing alert burden and need for guidance on standardized improved management approaches [6,31,32]. Recently, an international expert consensus statement was created by the Heart Rhythm Society and other large cardiac organizations to provide guidance for device clinics and clinicians on managing CIED follow-up, with some recommendations on operationalizing RM follow-up; however, this guidance lacks specificity on how to react to clinical issues detected via CIED RM [1]. In this study, some clinicians reported that their respective hospitals had instituted internal protocols for managing RM data, yielding positive outcomes in workload management without compromising patient care. Given the clinician’s desire and potential benefits of a standardized approach to RM data management, improved clinical guidance on RM data management is required.

Insufficient post-CIED implant education was a key barrier identified across stakeholders. This study identified that many patients believe they do not receive adequate information, both peri-implant and upon discharge. This is in line with previous studies that have identified that patients have a substantial deficit in their CIED knowledge, despite having a strong desire to receive more information, specifically around restrictions on daily living and how to deal with device-related issues [8,9,33]. Clinicians noted that limited understanding of the technology by the patients can prevent the uptake of the RM service and increase patient anxiety living with a CIED. Despite this concern, clinicians noted that patient education is not enforced nor standardized, with variation seen in the provision of information due to factors such as CIED type, insurance status, CIED manufacturer, and clinic staff availability. Large language models show potential in addressing gaps in patient education for general cardiac risk factors [34]; however, further training is needed before clinicians can trust their ability to enhance understanding and engagement for patients with CIED [35]. Future co-design studies with key stakeholders are required to develop an effective and efficient program to allow adequate and standardized patient education, without significantly increasing clinician workload.

Finally, patient engagement with CIED management emerged as a prominent theme across stakeholders. CIED RM has the unique opportunity to better engage patients with their CVD management through the frequent transmission of cardiac data. Clinicians outlined that a future goal for RM is to better engage patients with the alerts received, through early contact on “actionable alerts.” A potential modality proposed by stakeholders for this engagement is through a digital tool such as an SMS text messaging platform or app, where patients could access their data or alerts and communicate with their health care team. Clinicians had mixed beliefs on the utility of data sharing with patients, with some believing that it would positively increase engagement, while others are concerned it would increase patient anxiety and clinic workload. Patients believe that if data or alerts were to be provided to them, it would need to be presented in a user-friendly format. Previous studies focusing on CIED RM data interoperability with patients found that the data shared should be simplified, yet informative [36], be personalized and accompanied with informational support [37], and can ultimately enhance shared decision-making without increasing clinical workload [38]. Whilst CIED data sharing with patients may improve patient management, the feasibility of this technology is yet to be thoroughly explored.

### Strengths and Limitations

The strength of this study is the involvement of both patients and multidisciplinary clinicians, thus providing a comprehensive perspective of CIED RM barriers and enablers. The study also mapped the elicited themes and subthemes to behavior change techniques, which can be used to target actionable strategies for future adaptations to improve the RM service. However, this study has some limitations that need to be considered. First, participants were only recruited from New South Wales, Australia, with most included patients located in metropolitan and regional areas. However, the included multidisciplinary clinicians also serve patients from rural and remote regions and thus have a strong understanding of the barriers and enablers of the RM service in these areas. Second, the approach to participant recruitment used convenience sampling, which may limit the generalizability of our results. Despite this, the participant population sampled is varied in their backgrounds, with patients having a wide spread of CIED types and indications for CIED implants, and clinicians having an appropriate mix of genders, occupations, and subspecializations for cardiologists. Thereby, the collected information is insightful and likely applicable to the wider population when informing future research and clinical directions of RM.

### Conclusions

This study highlights the benefits and challenges of CIED RM from the perspectives of patients and multidisciplinary clinicians. It emphasizes both the role of the patient with themes centering on patient engagement, education, and benefits, as well as that of multidisciplinary clinicians challenged by the wealth of data, alert burden, and complexity of tasks arising from RM. The findings can serve as a roadmap to action to guide the continued development and implementation of RM services into the future. It seems clear that there is great potential for patient and health system benefits from the implementation of good systems for RM, but we are not there yet.

## Supplementary material

10.2196/67758 Multimedia Appendix 1 Participant interview guides.

10.2196/67758 Multimedia Appendix 2 Subthemes, codes, and participant quotes.

## Abbreviations

CIED: cardiac implantable electronic device
COREQ: Consolidated Criteria for Reporting Qualitative Research
CVD: cardiovascular disease
RM: remote monitoring
TDF: theoretical domains framework
