# Supplementary material for: Barriers and Enablers to Routine Clinical Implementation of Cardiac Implantable Electronic Device Remote Monitoring in Australia Among Cardiologists, Cardiac Physiologists, Nurses, and Patients: Interview Study
Source: JMIR Cardio. 2025 Jul 18;9:e67758. doi: 10.2196/67758 (PMC12296206; doi:10.2196/67758)
Supplement: Multimedia Appendix 1 [file cardio-v9-e67758-s001.pdf]

THE UNIVERSITY OF SYDNEY

Cardiac Technician Semi-Structured Interview and Focus Group Discussion guide

Socio-demographic information

- Age:
- Gender:
- Race/Ethnicity:
- Occupation (including sub-specialty + Place employment):

CIED remote monitoring

- Number of patients undergoing remote monitoring:
- Length of duration managing patients with CIED remote monitoring:

Remote Monitoring Experience

1. Explain the normal process of remote monitoring and contacting patients of issues

   Prompts:
   i. How is an issue detected/flagged?
   ii. What data points are used in monitoring?
   iii. Is this data available to clinicians on demand?
   iv. How long following an event is the patient notified?
   v. How are they notified?
   vi. What advice do you give them?
      - Is there a guide/template for this?
   vii. What happens following patient notification?
   viii. Are you involved at other points following notification?

2. Explain how CIED remote monitoring effects your current workload

   Prompts:
   i. Is there an increased workload? Why?
   ii. Is there a decrease in workload? Why?
   iii. For your change in workload is there a correlation with a change in patient outcomes? How?
   iv. Is there adequate resource allocation to CIED RM?
   v. Do you find there is sufficient guidance on RM to follow when there are events detected?
      i. What areas would benefit from clearer guidance?

3. How would you describe your experience with RM?

   Prompts
   i. What is your overall impression of the current RM system? Why
   ii. What aspects of RM currently do you find beneficial?
   iii. What aspects of RM currently do you believe are an issue
   iv. What is currently missing in the RM system?
   v. What would you like to see in future RM developments?

Patient engagement
Patient engagement is defined as "Actions individuals must take to obtain the greatest benefit from the health care services available to them".
Furthermore, it encompasses the partnership between patient and health professionals working in unison to improve health outcomes. It is important that the patient should be engaged at the level of their own care, this includes but is not limited to:

- Interaction with CIED device,
- Education on CIED and remote monitoring,
- Communication with healthcare team,
- Goal-setting with healthcare teams,
- Access to CIED device data.

The aim of engaging patients with their management of their device and condition is to improve shared-decision making management and have the patient work in unison with the healthcare team to improve the clinical outcomes, e.g., QoL, decrease symptom burden, hospitalisation and improve mortality rates.

4. What is your understanding of patient engagement?
5. How do you assist patients to engage with their device management?
6. How could you have more of an impact in improving patient engagement?
7. How do you provide education surrounding device to the patient?
   Prompts:
   i. How and at what stages of management
   ii. What resources did you provide?
   iii. Do you feel equipped to answer patient questions/concerns?
8. How do you believe patients should receive information/education in the future?
   Prompts:
   i. Modality of education
   ii. Frequency of education
9. How should patients be able to interact with their CIED Data?
   Prompts:
   i. Modality of access
   ii. Frequency of access

THE UNIVERSITY OF SYDNEY

Digital Support Program

A digital support program is an initiative where the patient and healthcare team work together to optimize patient cardiac management through a digital platform. This program will build-upon existing remote monitoring systems and patient engagement strategies to gather information on the functionality of the heart and patient symptom burden to optimally monitor the patient's condition and identifying early of any abnormalities. This will allow the clinician to promptly implement the optimal management strategies, improve patient understanding and interaction as well as providing regular 'check-ins' to ensure the patient understands their condition state and is complying with the prescribed management. This involves regular communication, routine CIED data collection through remote monitoring, providing education on the patient's condition state and providing access to patient data in aim of improving shared-decision making management.

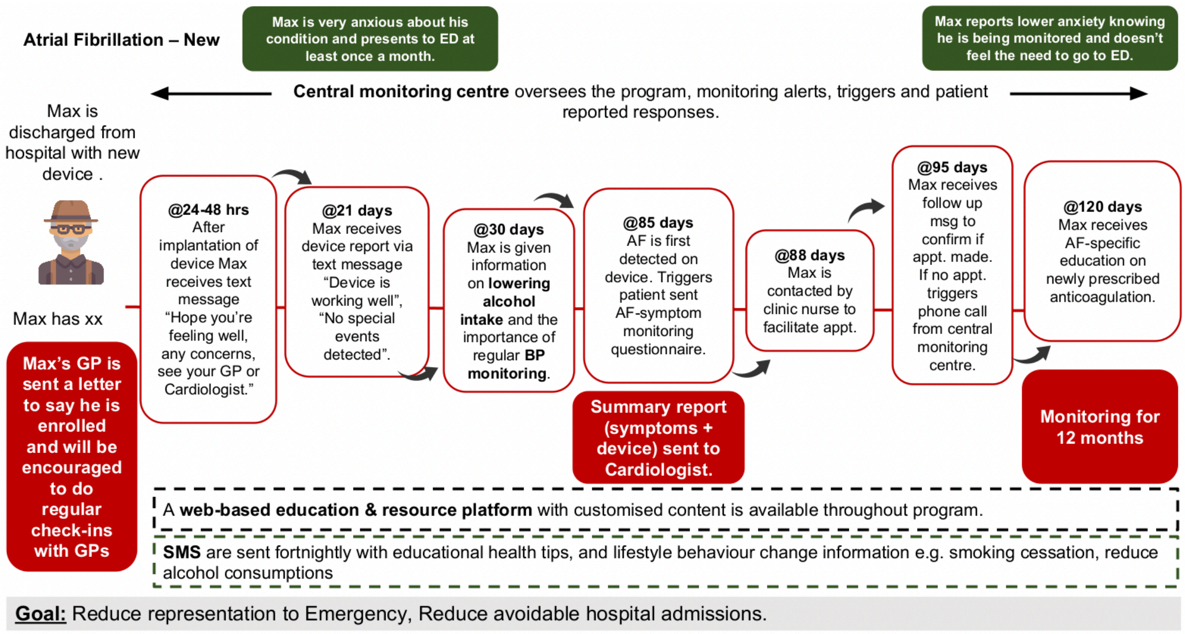

10. Are you open to the development of a post-CIED implantation patient digital support program? Why? Why not?

Clinician Semi-Structured Interview and Focus Group Discussion Guide

Socio-demographic information

- Age:
- Gender:
- Race/Ethnicity:
- Occupation (including sub-specialty + Place employment):

CIED remote monitoring

- Number of patients undergoing remote monitoring:
- Length of duration managing patients with CIED remote monitoring:

Remote Monitoring Experience

1. Explain your role in current CIED remote monitoring

   Prompts:

   a. How are you alerted of an issue?
   b. Do you have access to device data on-demand?
   c. How/when do you contact the patient following an event?
   d. How do you provide management following an event?
      i. E.g. virtually or F/U in clinic
   e. What happens following providing management advice?
   f. Is this an effective process?
   g. How can this process be improved?

2. Explain how CIED remote monitoring effects your current workload

   Prompts:

   a. Is there an increased workload? Why?
   b. Is there a decrease in workload? Why?
   c. For your change in workload is there a correlation with a change in patient outcomes? How?
   d. Is there adequate resource allocation to CIED RM?

3. From a clinician POV, how would you describe your experience with RM?

   Prompts:

   a. What is your overall impression of the current RM system? Why?
   b. What aspects of RM currently do you find beneficial?
   c. What aspects of RM currently do you believe are an issue?
   d. What is currently missing in the RM system?
   e. What would you like to see in future RM developments?

Patient engagement
Patient engagement is defined as "Actions individuals must take to obtain the greatest benefit from the health care services available to them".
Furthermore, it encompasses the partnership between patient and health professionals working in unison to improve health outcomes. It is important that the patient should be engaged at the level of their own care, this includes but is not limited to:

- Interaction with CIED device,
- Education on CIED and remote monitoring,
- Communication with healthcare team,
- Goal-setting with healthcare teams,
- Access to CIED device data.

The aim of engaging patients with their management of their device and condition is to improve shared-decision making management and have the patient work in unison with the healthcare team to improve the clinical outcomes, e.g., QoL, decrease symptom burden, hospitalisation and improve mortality rates.

4. How do you assist patients to engage in the management of their device and their condition?
5. How could you assist in improving current patient engagement?
6. How should patients receive information/education in the future?
   Prompts:
   a. Modality of education
   b. Frequency of education
7. How can we improve patient communication surrounding device/condition information?
8. How should patients be able to interact with their CIED Data?
   Prompts:
   a. Modality of access
   b. Frequency of access
9. How can clinicians improve goal-setting with patients?
   Prompts:
   a. Opinion on early discussion on aims of care
   b. Opinion on patient/Clinician agreement

Digital Support Program

A digital support program is an initiative where the patient and healthcare team work together to optimize patient cardiac management through a digital platform. This program will build-upon existing remote monitoring systems and patient engagement strategies to gather information on the functionality of the heart and patient symptom burden to optimally monitor the patient's condition and identifying early of any abnormalities. This will allow the clinician to promptly implement the optimal management strategies, improve patient understanding and interaction as well as providing regular 'check-ins' to ensure the patient understands their condition state and is complying with the prescribed management. This involves regular communication, routine CIED data collection through remote monitoring, providing education on the patient's condition state and providing access to patient data in aim of improving shared-decision making management.

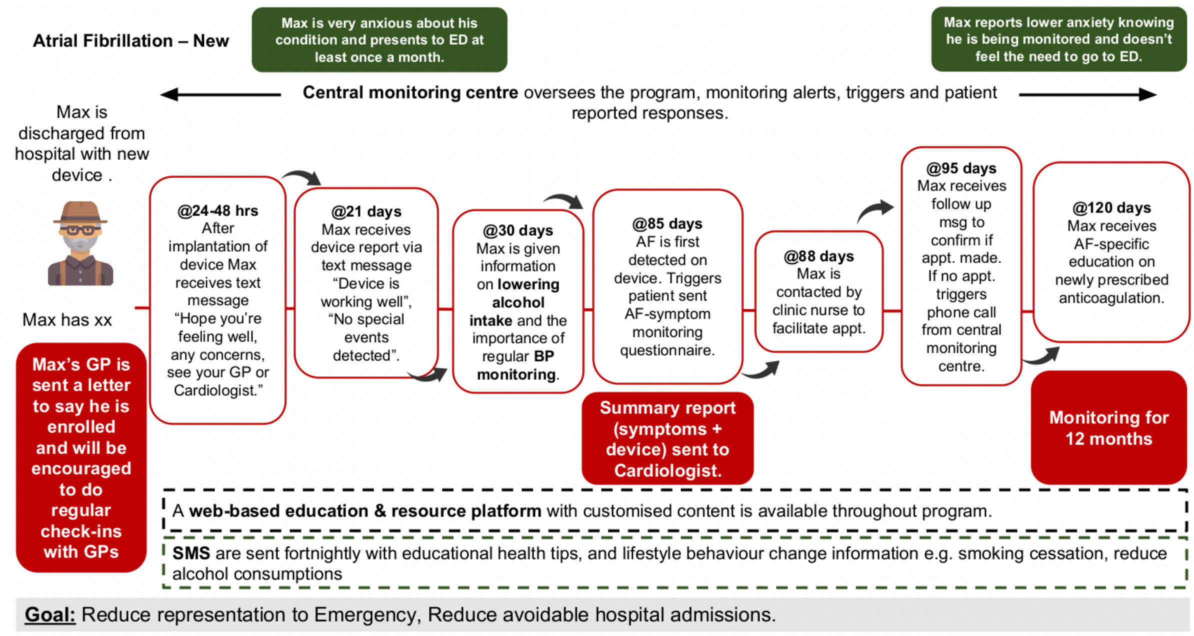

10. Are you open to the development of a post-CIED implantation patient digital support program? Why? Why not?

THE UNIVERSITY OF SYDNEY

Socio-demographic information

- Age:
- Gender:
- Race/Ethnicity:
- Occupation (including sub-specialty + Place employment):

CIED remote monitoring

- Number of patients undergoing remote monitoring:
- Length of duration managing patients with CIED remote monitoring:

Remote Monitoring Experience

1. Explain the normal process of remote monitoring when issues are detected?
   Prompts:
   i. How are you contacted of an issue?
   ii. What is your role when issues are detected?
   iii. When and how do you escalate your concerns?
   iv. What is your initial management when issues are detected?
   v. How do you follow-up with patients following initial management?
   vi. How are you involved at other points following event detection?

2. Explain how CIED remote monitoring effects your current workload
   Prompts:
   i. Is there an increased workload? Why?
   ii. Is there a decrease in workload? Why?
   iii. For your change in workload is there a correlation with a change in patient outcomes? How?
   iv. Is there adequate resource allocation to CIED RM?
   v. Do you find there is sufficient guidance on RM to follow when there are events occur?
      i. What areas would benefit from clearer guidance?

3. How would you describe your experience with RM?
   Prompts
   i. What is your overall impression of the current RM system? Why
   ii. What aspects of RM currently do you find beneficial?
   iii. What aspects of RM currently do you believe are an issue
   iv. What is currently missing in the RM system?
   v. What would you like to see in future RM developments?

Patient engagement

Patient engagement is defined as "Actions individuals must take to obtain the greatest benefit from the health care services available to them".

Furthermore, it encompasses the partnership between patient and health professionals working in unison to improve health outcomes. It is important that the patient should be engaged at the level of their own care, this includes but is not limited to:

- Interaction with CIED device,
- Education on CIED and remote monitoring,
- Communication with healthcare team,
- Goal-setting with healthcare teams,
- Access to CIED device data.

The aim of engaging patients with their management of their device and condition is to improve shared-decision making management and have the patient work in unison with the healthcare team to improve the clinical outcomes, e.g., QoL, decrease symptom burden, hospitalisation and improve mortality rates.

4. What is your understanding of patient engagement?
5. How do you assist patients to engage with their device and condition management?
6. How could you have more of an impact in improving patient engagement?
7. How do you provide education to the patient surrounding their device and condition?

    Prompts:

    i. How and at what stages of management
    ii. What resources did you provide?
    iii. Do you feel equipped to answer patient questions/concerns?
8. How do you believe patients should receive information/education in the future?

    Prompts:

    i. Modality of education
    ii. Frequency of education
9. How should patients be able to interact with their CIED Data?

    Prompts:

    i. Modality of access
    ii. Frequency of access

THE UNIVERSITY OF SYDNEY

Digital Support Program

A digital support program is an initiative where the patient and healthcare team work together to optimize patient cardiac management through a digital platform. This program will build-upon existing remote monitoring systems and patient engagement strategies to gather information on the functionality of the heart and patient symptom burden to optimally monitor the patient's condition and identifying early of any abnormalities. This will allow the clinician to promptly implement the optimal management strategies, improve patient understanding and interaction as well as providing regular 'check-ins' to ensure the patient understands their condition state and is complying with the prescribed management. This involves regular communication, routine CIED data collection through remote monitoring, providing education on the patient's condition state and providing access to patient data in aim of improving shared-decision making management.

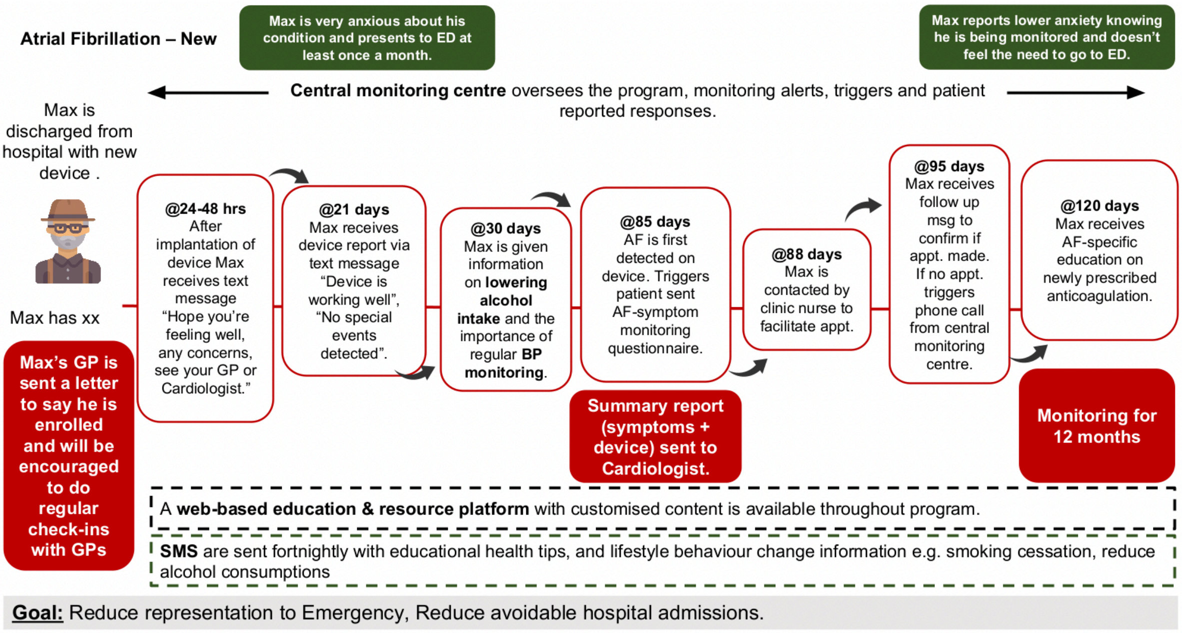

10. Are you open to the development of a post-CIED implantation patient digital support program? Why? Why not?

Patient Semi-Structured Interview and Focus Group Discussion Guide

Socio-demographic information

- Age:
- Gender:
- Race/ethnicity:
- Employment status:
- Education level:

Medical conditions and co-morbidities

CIED information:

- Device type:
- Device company:
- Indication:
- Implantation date:
- Duration of remote monitoring:

Remote Monitoring Experience

1. Explain your daily routine for remote transmission of your CIED data

   Prompts:

   i. How do you upload your CIED data?
   ii. How well do you daily adhere to data transmission?
   iii. How are you notified of failed transmissions?
   iv. How do you access educational information on your device?
      ▪ What resources are supplied to you?
   v. Who do you contact if you are concerned about your device?

2. How are you notified of issues with the device?

   Prompts:

   i. Who contacts you and how?
   ii. What information do you receive?
   iii. What is your overall impression of this process?
   iv. How could it be bettered?

3. Tell me about your overall experience with RM?

   Prompts:

   i. What is your overall impression of the current RM system? Why?
   ii. What aspects of RM currently do you find beneficial?
   iii. What aspects of RM currently do you believe are an issue?
   iv. What is currently missing in the RM system?
   v. What would you like to see in future RM developments?

Patient engagement

Patient engagement is defined as "Actions individuals must take to obtain the greatest benefit from the health care services available to them".
Furthermore, it encompasses the partnership between patient and health professionals working in unison to improve health outcomes. It is important that the patient should be engaged at the level of their own care, this includes but is not limited to:

- Interaction with CIED device,
- Education on CIED and remote monitoring,
- Communication with healthcare team,
- Goal-setting with healthcare teams,
- Access to CIED device data.

The aim of engaging patients with their management of their device and condition is to improve shared-decision making management and have the patient work in unison with the healthcare team to improve the clinical outcomes, e.g., QoL, decrease symptom burden, hospitalisation and improve mortality rates.

4. How are you actively engaging in your CIED remote monitoring?
   Prompts:
   i. Explain how you are currently engaging in this management, OR why are you not engaging?
   ii. How can we make improvements to allow you to be more engaged?
5. Explain how you received education surrounding your device and condition from your healthcare team?
   Prompts:
   i. Did this occur only at the start of your remote monitoring set-up?
   ii. What resources were provided?
   iii. What do you do if you have further questions?
6. How could the communication from the healthcare team (Technicians/clinicians) be improved?
7. How would you like to receive information/education in the future?
8. Would you find benefit in having access to your CIED data? Why/why not?
9. How would you like to interact with your CIED data?
   Prompts:
   i. Modality of access
   ii. Frequency of access
   iii. What data points would you like access to?

THE UNIVERSITY OF SYDNEY

Digital Support Program

A digital support program is an initiative where the patient and healthcare team work together to optimize patient cardiac management through a digital platform. This program will build-upon existing remote monitoring systems and patient engagement strategies to gather information on the functionality of the heart and patient symptom burden to optimally monitor the patient's condition and identifying early of any abnormalities. This will allow the clinician to promptly implement the optimal management strategies, improve patient understanding and interaction as well as providing regular 'check-ins' to ensure the patient understands their condition state and is complying with the prescribed management. This involves regular communication, routine CIED data collection through remote monitoring, providing education on the patient's condition state and providing access to patient data in aim of improving shared-decision making management.

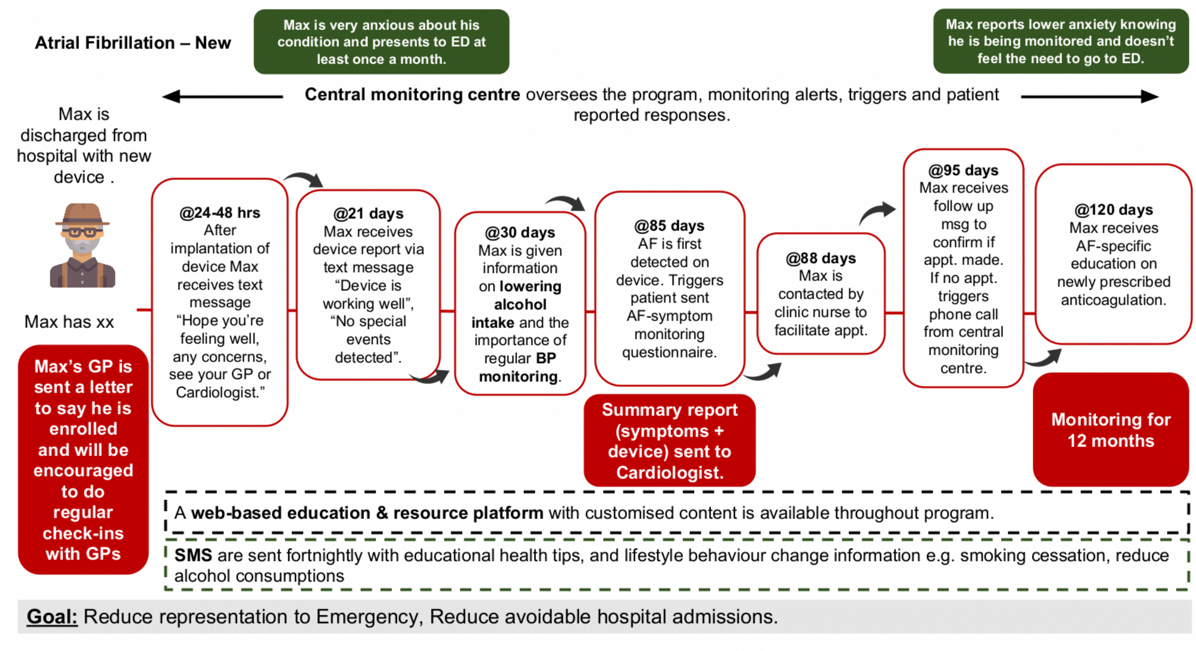

10. Are you open to the development of a post-CIED implantation patient digital support program? Why? Why not?
