# Supplementary material for: Barriers and Enablers to Routine Clinical Implementation of Cardiac Implantable Electronic Device Remote Monitoring in Australia Among Cardiologists, Cardiac Physiologists, Nurses, and Patients: Interview Study
Source: JMIR Cardio. 2025 Jul 18;9:e67758. doi: 10.2196/67758 (PMC12296206; doi:10.2196/67758)
Supplement: Multimedia Appendix 2 [file cardio-v9-e67758-s002.docx]

**Table S1:** Codes and quotes for the sub-theme ‘RM improves CIED and CVD management’

| **Sub-theme: RM improves CIED and CVD management** | | | |
| --- | --- | --- | --- |
| **Code** | **Barrier /Enabler** | **Quote number** | **Quote** |
| Improved patient treatment and outcomes | Enabler | 1 | “I mean, it’s only beneficial to us in the sense that it improves treatment for the patients” |
|  |  | 2 | “There’s no, no obvious benefit for us [Doctors] other than the fact that it improves patient outcomes I think.” |
|  |  | 3 | “I think it’s positive in that we’re able to pick up things and act upon it and provide a better service for our patients.” |
| Early detection of issues | Enabler | 4 | (Interviewee): “Well, you know when it’s happened on the spot, yeah. (Interviewer): “So early detection?” (Interviewee): “Oh yeah, without a doubt. Without a doubt.” |
|  |  | 6 | “We can get lead, we can get device malfunction and arrhythmia alerts early on.” |
|  |  | 7 | “The early notification of problems with the device or arrhythmias probably all are the main [benefits].” |
|  |  | 8 | “We often pick up things before the patient has an adverse clinical event. So you know we detect early signs of a lead failure and can act upon that before the patient has an adverse outcome because of lead failure.” |
|  |  | 9 | “We pick up a lot of arrhythmias and so forth which allow us to act upon before the patient has further clinical events or serious clinical events.” |
| Reduced post-implant issues | Enabler | 10 | “I mean we’ve both been here for a long time, well before remote monitoring and I remember clinics before remote monitoring and we used to have so much more troubleshooting, we’d have patients turn up that weren’t capturing, we’d have patients turn up – it seems like you never sort of knew what you were going to get whereas now it’s quite rare that we see a patient turn up with any significant lead issues like that.” |
|  |  | 11 | “In the first couple of weeks to see any [issues] you can pick that up a lot quicker if they have remote monitoring as soon as it’s implanted. |
| Prevention of hospital admissions | Enabler | 12 | “So we’re keeping patients out of hospital and actually doing a favour to the hospital.” |
| Providing service to rural/remote patients | Enabler | 13 | “I think it’s definitely beneficial for the patients. I think especially because we deal with a lot of rural patients, we’re really moving towards down the track trying just to be able to see a lot of those patients just by their remote monitor” |
| Avoided face-to-face reviews during COVID-19 | Enabler | 14 | “During COVID it was good too because you could do the same thing. We could still check the patients.” |

**Table S2:** Codes and quotes for the sub-theme ‘RM allows for an increased focus on patient-centred care’

| **Sub-theme: RM allows for an increased focus on patient-centred care** | | | |
| --- | --- | --- | --- |
| **Code** | **Barrier /Enabler** | **Quote number** | **Quote** |
| RM is user-friendly | Enabler | 1 | “I think one of the benefits that is that they don’t have to be [engage in data transmissions]…the whole point is that they put their monitor next to their bed and they forget about it” |
| Ability to provide service to patients unable to attend in-person | Enabler | 2 | “We’ve got a few patients that are in nursing homes. Them being able to just move over to remote care and not having to get someone to bring them down the hospital…means that their care’s probably a lot better than if we only had face-to-face” |
| Reduced in-person reviews | Enabler | 3 | “I didn’t mind having the home monitor, it meant me not having to go into the hospital as much” |
| Sense of safety | Enabler | 4 | “If anything goes wrong at home, they know about it, you don’t have to – you know, your doctors are hard to reach by phone so at least they might know about it”. |
|  |  | 5 | “I had an unsustained event. It was at 11:30 at night, I was fast asleep, I had no knowledge of it at all. But it is nice to know that it is being recorded, somebody looked at it and decided there’s nothing to be concerned about… Without remote monitoring, nobody would know about that at all, for some time.” |

**Table S3:** Codes and quotes for the sub-theme ‘Insufficient funding for management of RM service’

| **Sub-theme:** Insufficient funding for management of RM service | | | |
| --- | --- | --- | --- |
| **Code** | **Barrier /Enabler** | **Quote number** | **Quote** |
| Current reimbursement schemes are inadequate to ensure delivery of remote monitoring service | Barrier | 1 | “The main issue is a billing side of things. So because we are a public hospital and we don't charge the patients anything for remote monitoring is that we kind of really need to make sure that we are able to bill for it or we're always gonna make a loss from remote monitoring.” |
|  |  | 2 | “The main issue would be mainly the Medicare aspect. I think that that probably is the root cause of most of our issues. We could probably restructure how we work our clinics, especially in regards to loop recorder patients… depending on if the Medicare guidelines were different.” |
|  |  | 3 | “So probably the biggest headache is that the kind of guidelines with Medicare don’t actually reflect like the cardiac guidelines for actually following up patients.” |
|  |  | 4 | “It's a big burden of work [Alert monitoring] and I don't think there's any funding for it. So at the moment we’re all allocated onto other things like outpatient clinics or labs that sort of thing and we’ll quickly jump on remote monitoring in between patients and then if a patient arrives you shut down the remote monitoring and you see the patient.” |
|  |  | 5 | “But that whole process of the [Technician] detecting the issue, emailing me, looking up the notes to know that the patient’s never had AF, called the patient to see how they going then relayed all that to me. There's zero reimbursement for all of that work.” |
|  |  | 6 | “[Reimbursement] is probably the biggest headache and it comes up again and again with defibrillators, with Medicare, you can only bill once every six months like on the dot six months. |
|  |  | 7 | “Obviously with alerts there's no funding either, for it, which makes it a very expensive; but it is beneficial we’ve definitely seen benefits for our patients.” |
| Remote monitoring is provided with an excess cost to the clinics | Barrier | 8 | “So it costs us an overhead but at the end of the day, it gives us the best efficiency.” |
| Public hospitals are reluctant to cover the additional cost associated with remote monitoring | Barrier | 9 | “We raised it [implementing remote monitoring] at our cardiology department meetings at [Hospital] and outlined the benefits ,it's just the issue becomes the hospital does not want to outlay that cost.” |
| Lack of funding prevents the employment of adequate staff to manage alerts | Barrier | 10 | “If there was a reimbursement for every alert you checked, this would fund some of the man hours that are really necessary for remote monitoring, but at the volume you get now with just doing the scheduled checks, it's so time consuming and there’s not really the staff on paper allocated to remote monitoring because of the cost.” |
| Appropriate infrastructure and funding is required for remote monitoring delivery | Other | 11 | “I think funding is important. I think recognition by health services, that's really important to maintain staffing is really important. I don't think it's well recognised in a lot of health districts that it’s a critical requirement. To fund and recruit staff to run the service is actually quite labour intensive.” |
|  |  | 12 | “Unless [appropriate] infrastructure exists, unless government ends up paying for somebody to look at these things [Alerts] within the hospital system it's not going to progress at all.” |

**Table S4:** Codes and quotes for the sub-theme ‘Insufficient staffing for management of remote monitored alerts’

| **Sub-theme:** Insufficient staffing for management of remote monitored alerts | | | |
| --- | --- | --- | --- |
| **Code** | **Barrier /Enabler** | **Quote number** | **Quote** |
| Interpretation and actioning alerts can be time consuming | Barrier | 1 | “It definitely adds quite a bit of work. The techs that do it day-to-day, some are fast at it because they're more confident, others can take all day to get through their transmissions, which is fine, but it means that there kind of either later doing in the day because they’ve got other things to do on top or they literally have spent all day just doing remote monitoring.” (P17) |
| Inadequate staffing employed to manage workload | Barrier | 2 | “If a lot of our staff are on holidays then we're meant to be looking at those red alerts within I think a 48 hour period and sometimes we can’t actually do that.” |
|  |  | 3 | “If you say for example, school holidays or you got staff off sick, or for whatever reasons you couldn't get to it [alert monitoring] and, or especially on Monday you come where you’ve had alerts from Saturday, Sunday, Monday come through and then usually overburdened with a lot of things that there's a lot more to filter out. |
|  |  | 4 | “You need a certain critical number of people involved to monitor the data that’s coming in and to provide feedback for the patients going out. You need a fairly critical number of people to fill those spots to make sure there are no gaps during the week.” |
| Poor staffing prevents timely alert management | Barrier | 5 | “Sometimes when we're understaffed we get behind in looking at the remote monitoring. Sometimes the reports aren’t communicated in a timely way.” |
| Inadequate allocated time to review alerts | Barrier | 6 | “I think the technicians, their perspective they may say they would like a little bit more time, little bit more person hours to fulfill the role of checking alerts and checking routine 6 or 12 monthly scheduled remote monitoring checks” |
| Cardiologists don't dedicate time to review RM alerts | Barrier | 7 | “Disruptively interruptive is maybe one way of thinking about it as I don't have dedicated time to review remote monitoring or to receive updates on it, I just basically get called by people randomly when something comes through.” |

**Table S5:** Codes and quotes for the sub-theme ‘Remote monitoring access inequity’

| **Sub-theme:** Remote monitoring access inequity | | | |
| --- | --- | --- | --- |
| **Code** | **Barrier /Enabler** | **Quote number** | **Quote** |
| Not all CIED clinics provide patients with RM | Barrier | 1 | “Some patients who’ve moved into the area and previously had their device checked at the local hospital and they come in here and they’re [surprised], that [remote monitoring] hasn’t been provided in the hospital.” |
|  |  | 2 | “Patients should actually be able to get a remote monitor and currently through the public system they don't supply remote monitors. |
| Lack of funding has led to patient inequity receiving RM | Barrier | 3 | “Patients are essentially disadvantaged by not having remote monitoring because they’re having to come six monthly, the others only come 12 monthly; they don't have that closer monitoring. You're not treating the patients equally. So we have had discussions about whether there's room to, to absorb that cost and make it that if any patient eligible who is eligible under our criteria, that way they are given remote monitoring no matter what company.” |
|  |  | 4 | “You need free monitors for it to really work - that would just be a huge leap ahead if everyone gets a free monitor, you know you’d double your population or triple your population.” |
| Smaller cardiology clinics have limited capacity to provide RM | Barrier | 5 | “But a lot of the times these people go back to these little cardiologists’ rooms where they don't have the big facilities to actually monitor their patients or do not have the remote monitoring available.” |
| Device incompatibility can prevent remote monitoring uptake | Barrier | 6 | “So after their implant you need to check that they’ve got a device that's going to be compatible with remote monitoring because back in the day there - many pacemakers were not compatible with remote monitoring.” |
| Poor internet access prevents remote monitoring use | Barrier | 7 | “There has been issues in the country with people not having access to adequate capacity with some of the devices because of not having the wireless coverage.” |

**Table S6:** Codes and quotes for the sub-theme ‘Remote monitoring ‘Alert Burden’

| **Sub-theme:** Remote monitoring ‘Alert Burden’ | | | |
| --- | --- | --- | --- |
| **Code** | **Barrier /Enabler** | **Quote number** | **Quote** |
| Time consuming to review alerts | Barrier | 1 | “It’s probably less clunky than it was at the beginning. I feel like we’ve probably got rid of a lot of things that were causing us headaches, but it still takes up a lot of our time, especially, on a Monday morning you come back from a weekend of alerts and it's a lot to wade through, like you can spend the whole day doing it, just depending on what’s happened on the weekend.” |
|  |  | 2 | “You have to go through the process of checking it, so it is time consuming, even though we know generally it's 99.9% of the time going to be [non-actionable], but it all adds up having to check alerts |
|  |  | 3 | “The alerts is far more time consuming than the scheduled transmissions and we’ve spread our scheduled transmissions over the Monday, Tuesday, Wednesday. But alerts are definitely the larger part of the workload.” |
| Unable to modify parameters for all CIEDs | Barrier | 4 | “There is certainly some patients with inappropriate alerts that we’re unable to modify because of the way that those particular companies’ alert systems are set up. You can't just turn those alerts off, but we know that it's not actually relevant or it's already been addressed.” |
|  |  | 5 | “You know there’ll be alerts that you get daily and we check them anyway, but you almost always know it's not going to be actionable… You just can't turn them off in the system. |
| False alerts increase workload | Barrier | 6 | “A lot of those alerts aren't true alerts, like from loop recorders there's always false pauses, which takes up a lot of your time to look at when we could be looking at the real” |
| High workload compromises patient care | Barrier | 7 | “If it's a system where the alerts are just general alerts and it's the same alert set for every patient you're going to get overburdened with so many alerts where it’s, you'll be hard to filter out and get to the actual critical ones.” |
|  |  | 8 | “I don't think that's a problem with the software. I think it’s a problem with mismanagement on our part…. Missing crucial alerts, things like that.” |

**Table S7:** Codes and quotes for the sub-theme ‘Inconsistencies in interpretating and managing RM alerts’

| **Sub-theme:** Inconsistencies in interpretating and managing RM alerts | | | |
| --- | --- | --- | --- |
| **Code** | **Barrier /Enabler** | **Quote number** | **Quote** |
| Knowledge and experience influence alert interpretation and management | Barrier | 1 | “Different people have different knowledge and some will just kind of regurgitate what an automated report will say, and others have the insight to kind of look at - knowledge and experience really comes down to but - to look into it, understand it and to give it some clinical relevance.” |
|  |  | 2 | “We've been doing it for so many years now, feel like we've got through a lot of the [initial issues] and it’s quite a well-oiled machine at the moment.” |
|  |  | 3 | “I think knowledge and confidence is definitely a big thing. I think probably confidence in knowing that when you look at something, you're understanding it and you're not missing anything, is a big thing. And then I guess making unnecessary calls.” |
| Lack of guidance on alert management for inexperienced staff | Barrier | 4 | “I don’t think there’s a lot of guidance [alert management], but it's alright for us that have been working here a long time. New staff, it's so much harder for them because they don't understand it as much |
|  |  | 5 | “If you had a whole lot of people who are coming and going, you couldn't keep your staff, the yes, you’d need this very structured [approach] on what to do.” |
| Disparities between doctors on the management of alerts | Barrier | 6 | “A loop recorder that had documented pause episodes and [patient] sees an external cardiologist who's on holidays and has no one looking after his patients. So I asked two different advanced trainees. The first one I asked recommended that the patient contacts his own cardiologist and makes a follow up appointment and then the second one I asked, has requested the patient be called and come to ED.” |
| Disparities between doctors on what information physiologists provide them | Barrier | 7 | “It is doctor dependent [Contacting cardiologists] as well, so some doctors don't like to be told everything, whereas other doctors want to be told everything. So it’s, yeah, it’s tricky because it is quite doctor dependent.” |
| Discrepancy between baseline alert settings among CIED types | Barrier | 8 | “Devices have different sort of baseline settings and different sorts of alert thresholds and so forth. So there's a lack of standardisation.” |

**Table S8:** Codes and quotes for the sub-theme ‘Need for alert management guidance’

| **Sub-theme:** Need for alert management guidance’ | | | |
| --- | --- | --- | --- |
| **Code** | **Barrier /Enabler** | **Quote number** | **Quote** |
| Alert management guidelines will provide needed support | Barrier | 1 | “Now I do know that there are some guidelines I believe coming out that will support that sort of hands-off approach…Having the sort of guidelines there that say this is probably how it should be done I think will support people to have that change in practice.” |
|  | Other | 2 | “I think the biggest improvement would be if we did have a state or a national spreadsheet to go off… a uniform approach [to manage alerts].” |
| Identification of clinically relevant / actionable alerts only | Other | 3 | “Trimming the fat, so to speak, so getting clinically relevant information from all of the information that’s available [from RM], is a real challenge. There’s so much information there, what’s clinically relevant and what isn’t? So I think streamlining or more efficient systems that can facilitate your access to clinically relevant information is a really big challenge.” |
| Expert consensus panel to develop guidance on alert management | Other | 4 | “Consensus statements that you can get a steering group at least at a national level, perhaps you know the scope for future international level. [To develop] agreement on what would be the standard of care in terms of cardiac monitoring alerts and the type of alerts. |
| Decreased workload with alert management protocol | Enabler | 5 | “There’s a lot a lot less inappropriate emails being sent now. I think [technicians] workload has probably decreased as well, the amount of stuff that they just have to wade through that isn't really appropriate. So, yeah, makes it easier for us, not having to chase them up.” |
|  |  | 6 | “Since implementing the protocol there's been improvements in workflow… Less unnecessary notifications from our point of view.” |

**Table S9:** Codes and quotes for the sub-theme ‘Inadequate patient post-implant education’

| **Sub-theme:** Inadequate patient post-implant education | | | |
| --- | --- | --- | --- |
| **Code** | **Barrier /Enabler** | **Quote number** | **Quote** |
| Patient uncertainty on remote monitoring function | Barrier | 1 | “How does the home monitoring work? And is it daily? Do the reports get sent in daily? Because I guess in the early stages I was concerned that I’ve got a foreign body in my body, is it going to affect me daily, weekly, monthly? Should somebody be checking on me so regularly?” |
| Lack of explanation on patient restrictions | Barrier | 2 | “I think there’s a big gap in education for people that have had one [CIED] put in. I still don’t know what you can and can't do with it. I've got some trepidation.” |
|  |  | 3 | “Is it [CIED] going to limit your ability to drive a car or whatever for what period of time? What restrictions is it [CIED] going to put on your life immediately after the device is implanted, and if so, how long are those restrictions going to last? Will there be any permanent restrictions.” |
|  |  | 4 | “If I reach for something, I’ll use my right hand to reach up, I try not to use my left hand because I'm mindful of stretching and again I don't know how much tolerance there is in stretching, to affect things.” |
|  |  | 5 | “I was comfortable because I knew they were handing me a device that theoretically the battery was brand new. But again, uncomfortable in that I didn’t know what the limitations, if any, were on this one until I went home and read the instruction book.” |
|  |  | 6 | “I didn't know how easily these things would be dislodged. And I still don't know. I think people have a lot of questions and a lot of concerns particularly about is it safe to do this and what would it take for these things to become dislodged from my heart. I think they’re huge gaps on lifestyle stuff and there’s probably other people other worrying about whether they can do certain things,” |
|  |  | 7 | “I don’t know how many weeks after that you gotta keep your arm down for. I used to tie my arm down. This is again, the lack of information and people trying to do the best they can, make it up as they go along. I wear a belt and I’d tie my wrists to the belt So I wouldn't lift my arm in my sleep without knowing.” |
| Patient difficulties retaining information peri-implant | Barrier | 8 | “I can remember the information I got when the device was put in; I think you’ve got so many things going through your head. Reading stuff at that point, you know, there wasn't the opportunity to be able to really talk to someone and ask them questions. Specific questions relating to you.” |
| Discrepancy in education provision based on CIED type | Barrier | 9 | “A senior nurse who runs all the defibrillators…will give inpatients a sit down talk about defibrillators… But pacemaker patients won't really have any of that kind of, you know, deep and meaningful interaction.” |
| Discrepancy in education provision based on insurance status | Barrier | 10 | “In the private, I try to provide all the patients with the full disclosure from the device. So I give them more information than they understand. So it varies.” |
|  |  | 11 | “So, in the public it’s very much one off [Provision of education]. And then if there are issues down the track, then it's kind of putting out spot fires. So education as needed.” |
| Poor patient awareness of information resources | Barrier | 12 | “There's other things you'd like to be able to ask people about and I don't feel that people with pacemakers have got anywhere to go or any way of getting that information.” |
| Unable to ask specific CIED questions | Barrier | 13 | “I think they’re huge gaps on lifestyle stuff and there’s probably other people other worrying about whether they can do certain things with the pacemaker whatever, but there's been no one, nowhere I could go to get that information. And I certainly couldn't ask the technicians.” |
| No structured post-op support program for cardiac device patients | Barrier | 14 | “There’s not support for people once it's put in. Like it’s expertly put in. But there's nowhere to go [For information]. If you have a heart attack they’ve got a clinic in [Hospital], where you can go to get some counselling about heart attacks. The same thing doesn’t apply with information about the pacemaker.” |
|  |  | 15 | “I think with the pacemaker stuff people still have a lot of questions. I’ve still got questions. I don't know if I can dive into the water. I jumped into a gorge the other day and I'm thinking, should I have jumped in the water? I think there's so many lifestyle things that people don't know enough about 'cause there was no real extension programme, no one I could go and talk to after I had this thing inserted.” |
|  |  | 16 | “The patient doesn't know what that [CIED] looks like, they don't know if it's a plastic wire or metal wire or what even how it actually connects to the heart. So if you had a seminar where you can have bring some demos and say OK, this is actually what it is, this is how it works, this is how flexible it is and things like I think physical models will help the patient understand a lot more.” |
|  |  | 17 | “If we had someone that could kind of sit down, do an information session, I think that would probably make patients more aware of why we’ve given them a device, why we’ve given them a remote monitor. What that monitor really does, so they know, this is what [technicians] are going to be checking.” |

**Table S10:** Codes and quotes for the sub-theme ‘Patient education is limited by healthcare team availability’

| **Sub-theme:** Patient education is limited by healthcare team availability | | | |
| --- | --- | --- | --- |
| **Code** | **Barrier /Enabler** | **Quote number** | **Quote** |
| Discussions and explanations with patients are limited by clinician workload | Barrier | 1 | “I don’t think we give patients the opportunity [education]. I mean, we slam through here one every 15 minutes, if someone's gone into AF and they want to know what AF is, 15 minutes is not a long time to explain it properly.” |
|  |  | 2 | “As a cardiologist one-to-one you’ll be told this many times, we don't actually have enough time to do proper education” |
| Lack of explanation of CIED and condition during scheduled reviews | Barrier | 3 | “Well, when you go in and get it tested they don’t really explain what they’re doing. They put it on, talk to each other then say it’s OK, [They should] say we’re testing this and we’re testing that. You’ve got another five years left on your battery and stuff like that.” |
|  |  | 4 | “I think a lot of them [patients] want to know what the device is doing. I don't think we explain it well enough here, but I think a lot of them don't understand. We just say, yeah it's going great, no problems and then send them on their way.” |
| Limited access to healthcare team to ask questions | Barrier | 5 | “I could say the first 12 or 18 months I got the first pacemaker, nearly every time I saw Professor [redacted] I had a list of questions with me. And so I was waiting 6 months to get answers to the questions.” |
|  |  | 6 | “I wanted to take up flying model aircraft. They use a radio transmitter. I couldn’t find anywhere online that indicated whether or not the megahertz that they transmitted would interfere with the pacemaker. So I waited until I saw the technician when I was having a battery check, to ask him. He found out for me.” |

**Table S11:** Codes and quotes for the sub-theme ‘Patient anxiety associated with remote monitoring’

| **Sub-theme:** Patient anxiety associated with remote monitoring | | | |
| --- | --- | --- | --- |
| **Code** | **Barrier /Enabler** | **Quote number** | **Quote** |
| Patient privacy concerns | Barrier | 1 | “Some people, they hate the idea that anything is spying on them, they hate the idea you know they’ve often grown up in a world where you know literally the government is.” |
| Inconsistency of data information delivery | Barrier | 2 | “I got a phone call from [Doctor] office to tell me that the battery was failing in my pacemaker, that I needed to get to a hospital ASAP. So this is only a month after I was told, well, I had 3 ½ years left, and a month after I was told the first alarm was a false alarm.” |
| Anxiety with travel | Barrier | 3 | “When we travel in Australia, when we’re just driving anywhere, I make sure I'm somewhere near a hospital. It’s ridiculous. I mean, I think it’d be just bad luck. But, you know, I’ve got a wife who’s concerned that these devices are not reliable.” |
|  |  | 4 | “My wife will not travel overseas with me because she is too concerned. If we get away from a hospital where they can do a quick replacement, she doesn’t want to go. So, it’s buggered up our plans for travel.” |

**Table S12:** Codes and quotes for the sub-theme ‘Need to improve patient engagement with alerts’

| **Sub-theme:** Need to improve patient engagement with alerts | | | |
| --- | --- | --- | --- |
| **Code** | **Barrier /Enabler** | **Quote number** | **Quote** |
| Communicating with patient upon alert detection | Other | 1 | “There’s a huge gap where people need to be able get access and be reassured about whether this monitoring and the things are still working and implanted in their chest.” |
|  |  | 2 | “Knowledge that will change behaviour in a good way [should be provided], not knowledge that will change behaviour in a useless way.” |
|  |  | 3 | “Perhaps it's useful for the patient to be told [Alert detection] so that then they can correlate that with what their behaviour has been, for example maybe that they drink too much.” |
| Patient notification on actionable events only | Other | 4 | “I’m happy not to know that there’s not a problem. There’s no point in being told of an event that’s not going to affect me in one way or another. If there’s an event that affects me somebody should contact me and say.” |
|  |  | 5 | “If everything’s going well, I think you wouldn’t [Notify patients] but if there was things going on you’d want to know. I don’t know if you’ve ever been defibrillated, even if you’re awake, it’s not pleasant.” |
| Digital platform for patient communication | Other | 6 | “A lot of the time we have to call patients, they might not be at home, we're leaving a lot of messages. And you’re trying to track them down to figure it out. That takes up a chunk of our time too. So if you have something where they're able to tell you why they’re having those symptoms and instead of us having to, you know constantly try and call them that will probably be an easier, make our job a bit more streamlined.” |
|  |  | 7 | “That would certainly help [communication portal], I don't know anybody here. So when I get AF, if I get it bad, I just come either here or to [Hospital].” |
|  |  | 8 | “I think if there was some kind of text messaging service where if they had an issue and then we could just, like write back saying yes it's OK or no call the clinic. I think something very simple like that would be better than them having a lot of information.” |

**Table S13:** Codes and quotes for the sub-theme ‘CIED data sharing with patients’

| **Sub-theme:** CIED data sharing with patients | | | |
| --- | --- | --- | --- |
| **Code** | **Barrier /Enabler** | **Quote number** | **Quote** |
| Patients should have access to their data | Enabler | 1 | “I agree that patients should have access to data and where they request it there should be no reason why they shouldn’t receive that data.” |
| Personalised data provision can improve engagement | Enabler | 2 | “There’s a lot of fitness tracking information on the devices which I think is underappreciated and would help with engagement. I think being able to provide them more information from the device which is available, but we don't do, I think would improve engagement” |
|  |  | 3 | “I think when patients get provided instantaneous information and feedback and it's not just a box next to the bed - I think patients are much more likely to be engaged.” |
| CIED data sharing could increase patient anxiety | Barrier | 4 | “I don't know if the patient being able to see data is necessarily a good thing…. I think it would probably cause anxiety for some patients” |
|  |  | 5 | “It could be a very bad thing [data sharing]. Because they’re not trained to interpret the data. So if you give them too much then they’ll just end up calling with a whole lot of questions” |
|  |  | 6 | “I think it’d be a two-edged sword. While I think they'd be happy if they could look at it and say, yeah, everything’s OK, that's great. If you say your burden has increased, you'll have a phone call and a panicked patient here really, really, really quickly.” |
| Careful consideration would need to be taken if sharing CIED data with patients | Enabler | 7 | “You would have to be very careful what data you gave and how you presented it, and what you withheld.” |
| Data provision would need to be user-friendly | Other | 8 | “If people can access it [Data], it has to be a sort of an idiot ‘s guide for people to be able to make sense of it. It’d have to be in a way that they can somehow in a very rudimentary way, understand if there’s a major problem or not.” |
|  |  | 9 | “It’s also got to be very simple [Data report]. Generally, a large older population are still very computer literate; it [Data report] would have to be quite user friendly.” |
